# “I wish someone else could notify the partner”: barriers to STI partner notification identified and experienced by young women and health care workers in Cape Town, South Africa

**DOI:** 10.3389/frph.2025.1640282

**Published:** 2025-10-03

**Authors:** Fiona Bennin, Siyaxolisa Sindelo, Nomsa B. Mahlalela, Alison Buttenheim, Teniola Egbe, Prisca Vundhla, Pamela Fuzile, Mbali Jonas, Preethi Mistri, Brendan Maughan-Brown, Elzette Rousseau

**Affiliations:** ^1^ Desmond Tutu HIV Centre, University of Cape Town, Cape Town, South Africa; ^2^ Health Economics and Epidemiology Research Office (HE2RO) Wits Health Consortium, University of the Witwatersrand, Johannesburg, South Africa; ^3^ Center for Health Incentives & Behavioral Economics Department of Medical Ethics & Health Policy, University of Pennsylvania, Philadelphia, PA, United States; ^4^ Southern Africa Labour and Development Research Unit, University of Cape Town, Cape Town, South Africa

**Keywords:** partner notification, sexually transmitted infections, adolescent girls, young women, behavioral design, South Africa, NUDGE framework, co-design

## Abstract

**Background:**

South African clinical guidelines for sexually transmitted infections (STI) treatment and management recommend that all individuals who test positive should receive a notification slip to pass on to their partners. Despite these guidelines, partner notification and treatment rates remain low. Barriers include misinformation, gendered beliefs, and interpersonal concerns such as fear of stigma, violence, and being blamed for infidelity.

**Material and methods:**

We used a behavioural design approach to explore challenges experienced by adolescent girls and young women (AGYW) in notifying their predominantly asymptomatic male partners about an STI diagnosis. A total of 7 AGYW and 8 Health Care Workers (HCWs) participated in behavioural mapping and co-design workshops in Cape Town, South Africa. Insights and solutions for partner notification were identified using the behavioural science NUDGE theory framework.

**Results:**

Participants experienced various emotions when receiving a positive STI result, including denial, confusion around mode of transmission, fear of the impact on their future, as well as anxiety around their partners’ reaction. HCWs noted AGYW's limited understanding of STIs and challenges in communicating the diagnosis to their partners, particularly when one or both partners were asymptomatic. Both groups criticized the current partner notification slip as overly complex and legalistic. Suggestions included simplified slips, and approaches that minimize AGYW's role in partner notification.

**Conclusions:**

Our results provide insight into the barriers experienced and identified by AGYW and HCWs, from AGYW receiving a positive STI test result, through notifying their partners. Next steps involve developing and testing high-fidelity prototypes that reduce the burden on AGYW and are feasible for integration into standard clinical care.

## 1 Introduction

The prevalence of sexually transmitted infections (STIs) in South African young people is significant, with an estimated 5.0% for gonorrhea, 17.9% for chlamydia, 5.4% for trichomoniasis, and almost a quarter (23.7%) for any STI (1). South African clinical guidelines for STI treatment and management recommend that all patients testing positive for an STI, receive a partner notification slip for their sexual partners. As many STIs are asymptomatic, partner notification is important for preventing re-infection and curbing transmission (2, 3). The slip should specify the STI name and detected pathogens (4). However, despite these guidelines, the effectiveness of partner notification remains limited (5). A review of studies in Southern Africa showed that only 53% of index cases successfully notified partners, and of these cases, only 25% of those partners sought treatment (6). Furthermore, many male partners in Southern Africa avoid health facilities altogether, with up to 50% not following up for treatment (7).

Barriers to partner notification include limited health education/literacy, gendered beliefs that blame women for transmission, misinformation about STI causes (e.g., self-generating or poor hygiene or toilet seats), and interpersonal barriers such as fear of stigma, infidelity accusations, or violence (8, 9). Many adolescent girls and young women (AGYW) accept the partner notification STI slip without intending to notify their partner, either due to fear or lack of communication skills about STIs (5).

Alternative strategies such as Expedited Partner Therapy (EPT), where patients deliver treatment to their partners, have shown promise in other countries (10). Similarly, although not commonly used for STI treatment, courier delivery of treatment has been found to be a convenient and discreet method, seen in similar settings with Pre-Exposure Prophylaxis (PrEP) treatment delivery (11). However, the lack of counselling for the partner often results in communication of the diagnosis and explanation thereof, still falling on those initially diagnosed. While these (EPT and courier) are not yet widely used in South Africa, interventions like partner notification counselling and education have shown improved notification rates by 10% compared to standard of care (SOC) (12). This highlights the potential for a variety of partner notification strategies to be used based on the characteristics of the setting and patient/client.

The aims of this study were to explore the barriers and facilitators to STI partner notification and to explore potential interventions to increase partner notification and treatment rates using the NUDGE (Narrow, Understand, Discover, Generate, Evaluate) behavioural design framework (13) with a group of AGYW and Health Care Workers (HCW) from Cape Town, South Africa.

## 2 Materials and methods

### 2.1 Study setting and participants

This study was embedded in the FastPrEP study, an implementation science project, which offers PrEP (oral, vaginal ring, and injectable Cabotegravir) to adolescents and young people (15–29 years old) in Cape Town, South Africa. A purposive sample was recruited from a sub-study of FastPrEP, which aimed to determine the impact of standard of care (a trained nurse identifying STI syndromes based on participants reporting symptoms, then being treated according to South African national guidelines) compared to GeneXpert STI testing on the uptake of PrEP (4, 14). Following their STI diagnosis, participants in this sub-study would have been provided with information from a health care worker (HCW) regarding their specific STI, particularly on transmission, treatment and potential for re-infection if their partner was also positive but untreated. The participant would have been provided with a notification slip to give to their partner which encouraged the partner to attend the clinic to get treated for an STI (even if asymptomatic) and included information regarding what STI the person tested positive for and what treatment was given, in 3 South African official languages.

Participant IDs who met the inclusion criteria were extracted from the database and each participant was invited telephonically to participate in the study by a research assistant, until the sample size was reached. Participants were included in the study if they were 1) females between the ages of 15 and 29 years old who, 2) were part of the FastPrEP-STI study and 3) had received a STI positive result; as well as HCWs working on the FastPrEP-STI study (14).

#### Ethics statement

This study was approved by the University of Cape Town's Health Science Research Ethics Committee (233/2023) nested in the larger Fast-PrEP study (713/2021). Written informed consent was obtained from all participants for workshop participation, audio recording and for non-identifiable photos to be taken. All AGYW were reimbursed for their participation and all participants (AGYW and HCWs) were provided with refreshments.

### 2.2 Study design

**Figure 1:**
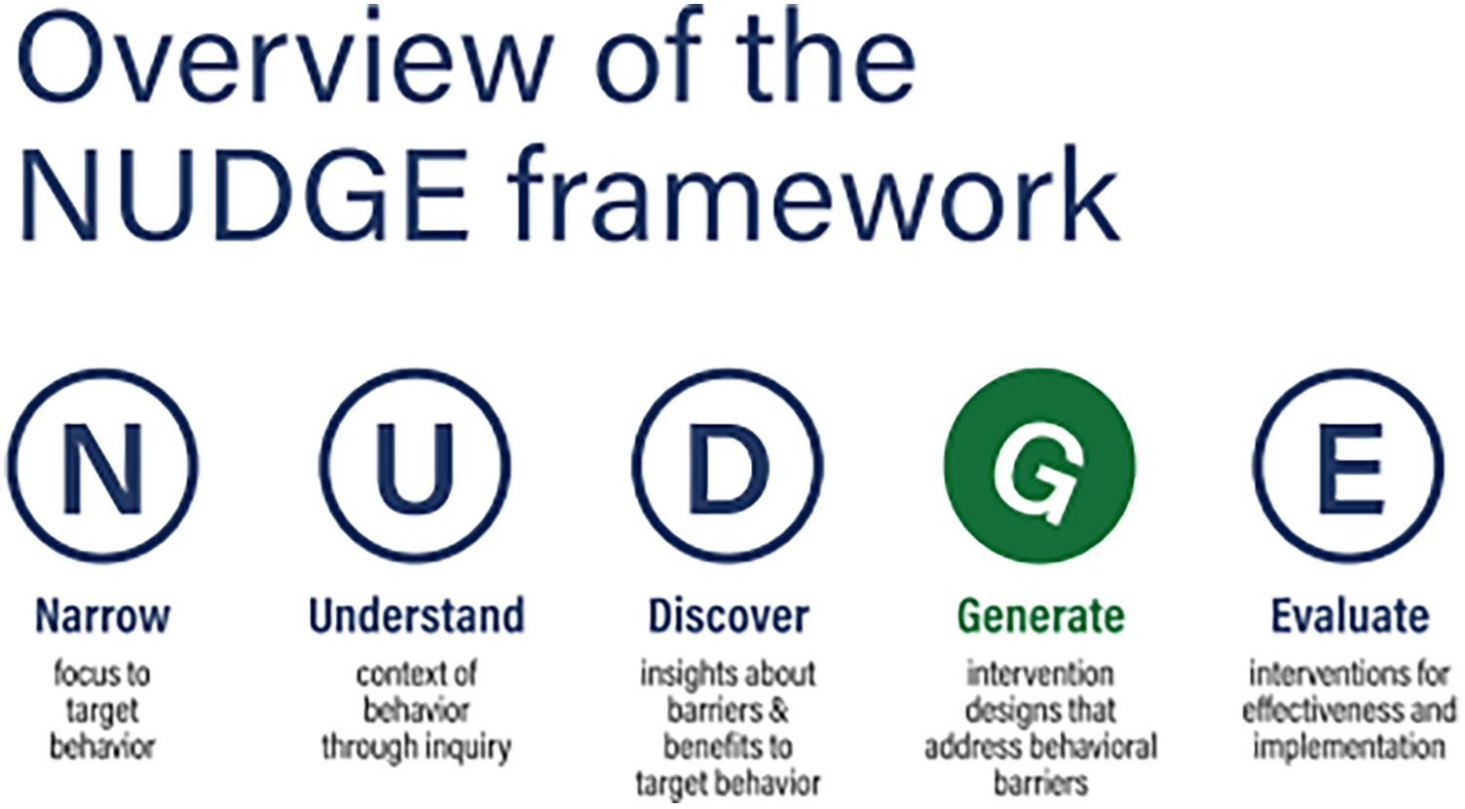
5-step NUDGE behavioural design framework (13).

We used the NUDGE behavioural design framework to identify partner notification-related challenges experienced by AGYW when notifying their predominantly asymptomatic male partners. NUDGE draws on behavioural design frameworks, design thinking and intervention mapping frameworks by encouraging researchers to proceed systematically through five steps of intervention design, including Narrow, Understand, Discover, Generate, and Evaluate (13) (see Figure 1). This approach was chosen as it focuses on discovering behavioural insights about the barriers being researched and generates solutions that incorporate behavioural solutions (13). As we wanted to determine both the barriers to partner notification as well as what AGYW and HCW would like the partner notification process to look like, the NUDGE approach was best suited to achieve this outcome. We describe the first four steps here.

A team of 11 researchers from HE2RO (*n* = 3), the University of Pennsylvania team (*n* = 2) and Desmond Tutu Health Foundation (DTHF) team (*n* = 6) led the workshops. A group of 7 AGYW and 8 HCWs attended two days of behavioural mapping and co-design workshops in March 2024. Each day consisted of two parts, namely morning preparation with the research team and afternoon workshops with the AGYW and HCW participants.

### 2.3 Design process

#### Day 1: contextual inquiry (narrow, understand, discover)

The aim of the first day of the workshops was to complete a contextual inquiry into the partner notification journey. In the morning of day 1, the research team *narrowed* the problem to the specific “focal” behaviour, clarified roles and practiced the prompts and note taking for the workshop (13). The DTHF team also rehearsed the sense checking session (checking in with other team members post workshop to compare notes and agree on major themes discussed), journey mapping [mapping the AGYW's journey from STI diagnosis to notifying their partner (or not)], and the iterative prototyping process (explained below under Day 2). In the afternoon, the research team was joined by the AGYW and HCWs to *understand* the context of partner notification through inquiry (13). Working separately (i.e., one group with all HCWs and the other group with all AGYW participants) to encourage frank and open conversation, each group discussed the facilitators and barriers to AGYW notifying their partners about their positive STI result, through the process of journey mapping. Both groups were encouraged to discuss the facilitators and barriers using a pre-determined set of prompts, to guide the discussion. Both the AGYW and HCWs were then asked to role play scenarios in which a) the AGYW needed to notify their partners about their STI, and b) the HCWs had to counsel an AGYW about their STI diagnosis and how to communicate the result with their partner. Each group was shown the current partner notification slip provided as SOC and asked their opinion on its design and purpose. After the insights from these discussions were generated, the top 3 insights were reported back to the bigger group and the group members were asked for just one aspect of the discussion for the researchers to work on/fix. Once the workshops were concluded, the research team sought to *discover* insights about the barriers to partner notification by distilling the output from the workshop into 5–8 key behavioural barriers that had potential to be addressed through interventions in which DTHF could implement (13).

#### Day 2: prototype co-design (generate)

The aim of the second day of the workshop was to *generate* intervention strategies that addressed the behavioural barriers by developing several prototypes which could be further refined and tested in a real-world setting (13). In the morning of day 2, the research team prioritised key behavioural barriers further and developed low-fidelity (first draft/iteration) prototypes. 3 prototypes were chosen to present to the groups in the afternoon workshop, which included key changes to the partner notification slip/process. In the afternoon workshops, all the participants and researchers were mixed and divided into three groups to discuss and further develop one prototype each. Each group revised, iterated on the prototype to incorporate suggestions and created another “higher fidelity” version of each prototype. Lastly, each group “pitched” their revised prototype to the full workshop group, with the potential to receive any further feedback from the other group members.

### 2.4 Data management and recording

The initial group discussions with the AGYW and HCWs were audio recorded, and notes were taken by two note takers, respectively for both groups. All notes regarding understanding the phenomenon and gathering insights were written on colour coded sticky notes which were then recorded in an excel document, which was fact checked and verified by all team members involved. Photos of the sticky notes were also taken as an additional source and added to the excel sheet for further reference.

## 3 Results

### 3.1 Participant characteristics

As each AGYW participant was part of the STI sub study, they would have a) received treatment and counselling about their STI diagnosis, and b) been provided with a partner notification slip for their partner/s. The average age of the AGYW participants (*n* = 7) was 26 years. Of the 8 HCWs, 2 were male, and 6 were female, with an average age of 34 years. 3 were nurses, 3 were counsellors and 2 were peer navigators.

### 3.2 AGYW and HCW insights & barriers to partner notification

After gathering the insights from the two groups, the main barriers identified could be grouped as understanding of STI diagnosis and treatment, partner notification counselling, and notifying the partner. The relevant quotes describing the respective barriers are included in Table 1.

**Table 1:** Quotes describing barriers to partner notification.

STI diagnosis	STI treatment and PN counselling	Partner notification
• “I don't think I have an STI, I have no symptoms” (AGYW) • “I don't believe I got this infection from my partner (e.g., toilet seat)” (AGYW)	• “I'm scared my partner will blame this on me” (AGYW) • “The PN slip indicates the specific STI—that's my personal information” (AGYW) • “The PN slip is too complicated, too much text, sounds legal” (HCW) • “I don't have the right tools to counsel the patient on PN” (HCW)	• “I don't know how to start this conversation with my partner” (AGYW) • “I don't feel like I can convince my partner to go get treatment” (AGYW) • “I wish someone else could notify the partner” (HCW)

Both the AGYW and the HCWs discussed the AGYW's limited knowledge around what an STI is, how it is transferred and how it presents itself (or does not i.e., with no symptoms). Many young people did not understand STI terminology and symptoms and had concerns about STI treatment efficacy. Similarly, the AGYW were concerned about how STIs and treatment thereof may impact their fertility. The AGYW reported that they do not receive counseling around STIs or guidance from HCWs on how to inform their partner, and this can lead to a lack of motivation in wanting to notify their partners. They were also concerned about their partners' reactions (which could lead to interpersonal violence or losing the partner), particularly if the partner is asymptomatic. Some young women were attending the clinic for other reasons such as family planning and said they did not expect an STI test and the subsequent result.

The HCWs mentioned that this often results in denial of the STI test results, which can make it difficult to counsel further or discuss a plan for partner notification. Furthermore, some of the HCWs reported that they did not feel equipped to counsel participants beyond the STI diagnosis, explaining that they wanted to have the “right” conversation with participants based on their individual history and current situation e.g., occasional vs. fixed partners, risk of intimate partner violence (IPV), etc.

All of the AGYW and HCWs discussed aspects of the partner notification slip that they did not like, or they felt could be improved. Both groups thought that the partner notification slip was too long, too intimidating and looked like a legal document, i.e., not something that someone would want to receive or give to their partner.

Some did not like the inclusion of all three local languages on the document (making it more text heavy) and suggested having each language on a different document. Lastly, some of the AGYW were hesitant about some of the information placed on the partner notification slip, noting that stating the STI on the document is a breach of their confidential information that they would rather not share with others.

The AGYW shared their apprehensions around notifying their partners. Some of the AGYW had no idea how to even start the conversation with their partner, with one of the participants wishing they could just leave the slip “on top of the TV” in the hope that the partner would see it and ask them about it, thereby initiating the conversation without the AGYW having to start it first. For those who were willing to initiate the conversation, some felt that they could not convince their partner to go for treatment, particularly to attend the clinic on their own. Others felt scared about how their partner would react, wondering if the partner would blame them and accuse them of cheating or even become violent.

Lastly, an important theme suggested the concept of removing the AGYW from the partner notification process altogether. As the majority of the HCWs shared the same concerns as the AGYW around partner notification, many felt that the burden should not lie with the AGYW to have the conversation, and it would be preferable if male partners could be contacted directly to initiate treatment themselves.

### 3.3. Prototypes and further suggestions

In the second workshop the researchers came up with three low fidelity prototypes to present to the group of participants. These were, 1) counselling aids, scripts and support for the HCW when counselling young people, 2) specific tools, support and strategies for AGYW to notify their partner (or take them out of the process) and, 3) modifying the partner notification slip (or invitation card). The participants discussed each suggestion and presented the following back to the larger group. At the end of the workshop the following prototypes were put forward for further development (see Table 2 for further details and photographic representation of each prototype):

1. A youth friendly, visually appealing invitation card (instead of the current partner notification slip)
2. An animation video, paper flip book or “zip zap” card (small card that can fit in a wallet/purse) containing relevant information about STI symptoms and treatment options.
3. SMS notification, which is sent to the partner.
4. A “super cool video” with STI information
5. Express medication given to the AGYW at the clinic to give to the partner later (EPT)
6. Some form of incentive for attending a clinic to get tested
7. Courier service sending the STI treatment directly to the partner

**Table 2:** Description and graphical representation of each prototype.

Prototype graphic representation	Prototype description
1. Invitation card 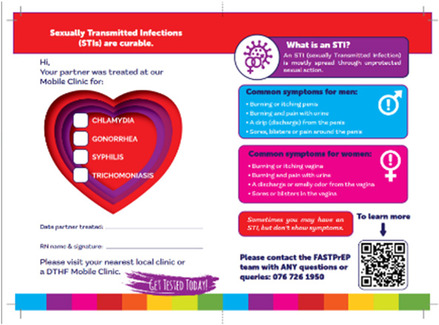	Youth friendly, visually appealing card which includes the same information as the original Partner notification slip, such as the name of the STI that needs to be treated, as well as other information about STI symptoms and transmission. The partner would take this to the clinic.
2. Paper flip book/Zip zap card 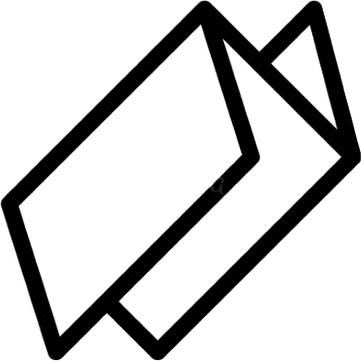	A small card that can fit in a wallet/purse, containing relevant STI information, that the HCW can go through with the AGYW and/or give to them to keep for future reference.
3. SMS notification 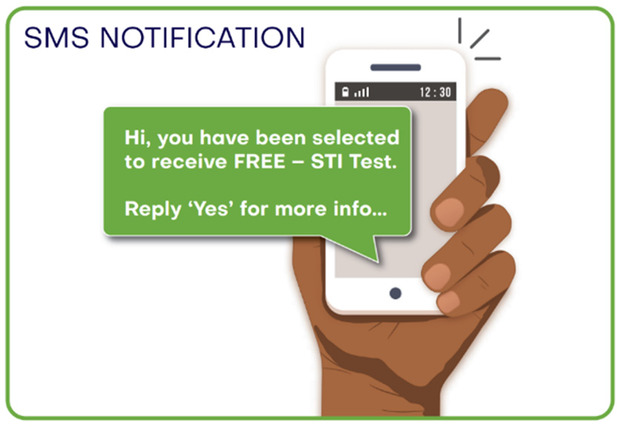	A SMS notification, which is sent to the partner, stating their partner has tested positive and inviting them to attend the clinic to be tested.
4. Animation video 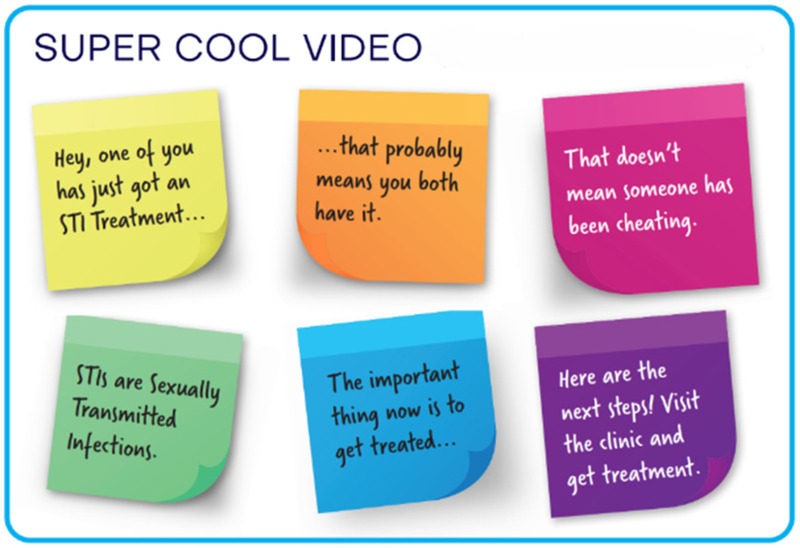	A youth friendly video with STI information that the AGYW could download or have the link for, to show to their partner at a later stage.
5. Express medication 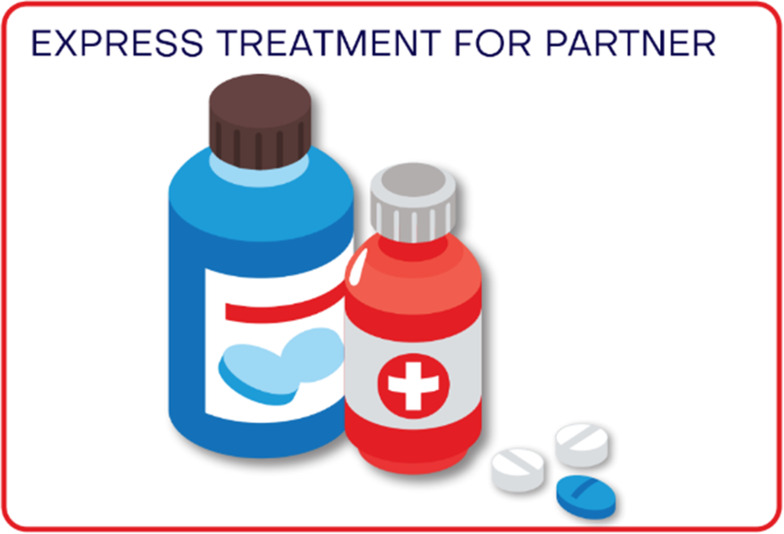	A pack of medication which is given to the AGYW at the clinic to give to the partner later (EPT) for treatment (she would receive the same treatment pack to take herself).
6. Treatment incentive 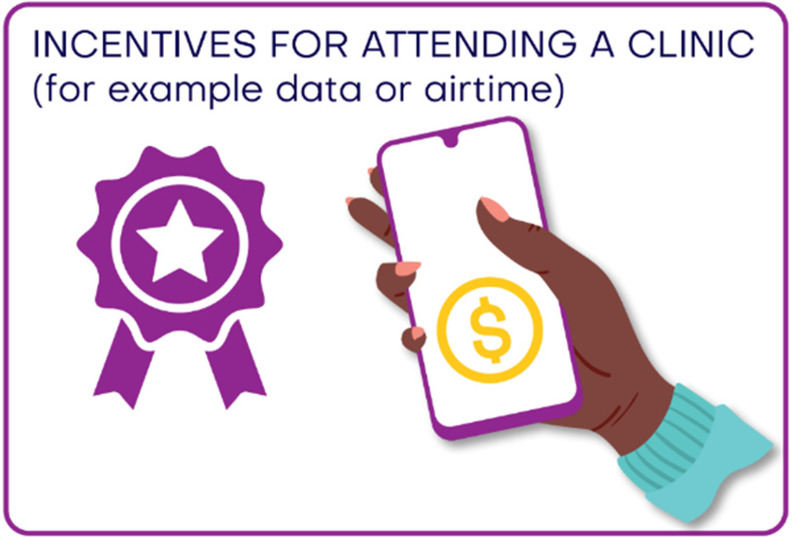	Some form of incentive for attending a clinic to get tested, such as a certain amount of data or airtime. This would be for the partner only, or for the partner and AGYW to attend together.
7. Courier service 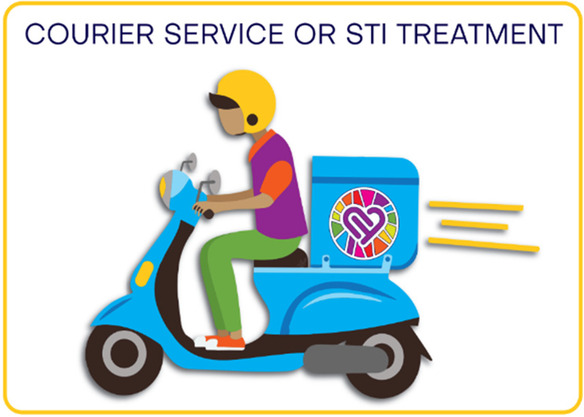	A courier service sending the STI treatment directly to the partner, without the AGYW involved. This requires the HCW to contact the partner to confirm delivery details.

## 4 Discussion

This study aimed to explore the barriers and facilitators of STI partner notification and treatment in a group of AGYW and HCW from Cape Town, South Africa. Both groups reported barriers around access to correct information, knowing how to inform partners and a dislike of the current partner notification slip. These barriers are commonly described in other similar contexts where young people have reported not having the resources or skills to persuade their partner to seek treatment, especially when notifying casual sexual partners, with many experiencing negative impacts on their relationships and reputation (5, 9). Health education barriers reported in this study remain an issue when notifying partners as young people do not have, or do not understand the basic information of how an STI is transmitted, often having misconceptions based on concerns around shared public toilets, lack of personal hygiene (especially in women), etc. (8).

The HCW in this study discussed how they do not feel equipped to have conversations about partner notification with AGYW and furthermore, how to equip the AGYW to notify their partners when they leave the clinic. Studies which have implemented partner notification training with HCW, recommend the provision of any training that promotes STI education and socio-emotional support for individuals with STIs who need to notify their partners (8, 15). However, despite counseling being a successful intervention for encouraging partner notification, there is limited evidence to suggest it decreases the annual incidence of STI diagnosis among people who have STIs (15).

One of the clearest messages from both the AGYW and HCWs was that STI notification and treatment should ideally remove the AGYW altogether, based on their suggestions/ideas to send partners a text/SMS inviting them to get tested or sending the medication directly to the partner via courier While EPT has been found to be successful in other countries and clinical trials, it is still not legal outside of the research environment in South Africa (16). However, while EPT is known to be successful, it still requires AGYW to physically give the medication to the partner. This can practically be difficult, especially if the young woman is not in contact with the partner anymore. While using a courier to send treatment to male partners does not take the AGYW out of the process altogether, it has been successful in this context with delivering PrEP for those who choose the service, making it a helpful choice for those wishing to be more discreet (11).

Three of the prototype suggestions that would be easier to implement include the updated youth friendly partner notification slip, the “zip zap” card and animated video. While pamphlets and other paper-based information can be helpful and have been reported to be effective in some contexts (17), there is always the potential for hard copies being lost or thrown away. While the video may have greater potential for engagement, any barriers to accessing online materials need to be removed i.e., data free, which can require substantial costs for the provider (18). Similarly, providing incentives for returning to the clinic for testing may not be feasible in low resource environments where resources are often not available to provide reimbursements. The next steps in this process will be to develop higher fidelity prototypes and conduct another series of workshops to receive further input from AGYW and male partners to determine the acceptability of each prototype and be aware of any contextual factors that may influence implementation of any new prototypes. Following this, a randomized controlled trial (RCT) will be conducted to determine which of the prototypes is most effective, acceptable and feasible in this setting.

This study has a few strengths and weaknesses that should be highlighted. One of the strengths of the study was the use of the NUDGE framework, which allowed, together with a co-creation approach, to find valuable insights into the barriers around STI partner notification, with the individuals in which it impacts the most. Having the AGYW and HCWs work together served as a strength and weakness, as it helped both groups to understand the other groups' viewpoint, however with regards to the AGYW, there may have been social desirability bias and potential for holding back in discussions, due to the HCWs being present. Lastly, while the process was meant to be rapid, it may have been beneficial to have had one more introductory session to brief the AGYWs about what would be discussed and how, to allow for maximum input on the topic, from the beginning of the workshop.

## 5 Conclusion

There are several barriers which deter AGYW from notifying their partners about their positive STI results, including limited knowledge of STIs, feeling ill equipped to having STI related conversations with partners, and the partner notification slip itself. Further development of high-fidelity prototypes, which ideally reduce reliance on AGYW to deliver partner notification partner notification, is required to find solutions for increasing treatment rates and reducing new STI infections in this population of AGYW and their male partners.

## Data Availability

The raw data supporting the conclusions of this article will be made available by the authors, without undue reservation.
